# Whole-Genome Sequences of 35 Incompatibility Group I1 Plasmid-Carrying Salmonella enterica Isolates from Food Animal and Clinical Sources

**DOI:** 10.1128/MRA.00831-19

**Published:** 2019-08-29

**Authors:** Nesreen H. Aljahdali, Pravin R. Kaldhone, Steven L. Foley, Bijay K. Khajanchi

**Affiliations:** a National Center for Toxicological Research, U.S. Food and Drug Administration, Jefferson, Arkansas, USA; b Biological Science Department, College of Science, King Abdul-Aziz University, Jeddah, Saudi Arabia; University of Arizona

## Abstract

We sequenced 35 Salmonella enterica isolates carrying incompatibility group I1 (IncI1) plasmids from different serotypes to study their genotypic characteristics. The isolates originated from food animals (*n* = 32) and human patients (*n* = 3). All isolates carried IncI1 plasmids, and many had additional plasmids detected along with virulence and antimicrobial resistance genes.

## ANNOUNCEMENT

Bacterial foodborne pathogens such as Salmonella enterica contribute to significant morbidity and mortality worldwide (1). Patients with more severe manifestations of illness often require antimicrobial treatment for resolution of disease (2). Unfortunately, several *Salmonella* strains are resistant to antimicrobial therapy, and often, this resistance is encoded on plasmids (3). Several plasmid types, including members of the incompatibility group I1 (IncI1), have been implicated in carrying antimicrobial resistance genes (4). Additionally, IncI1 plasmids have been reported to carry genes that may increase the virulence of strains where they reside (4). The aim of this study was to assess the antimicrobial resistance and virulence gene content of IncI1-positive *Salmonella*. We sequenced 35 Salmonella enterica isolates originating from cattle (*n* = 12, 34%), swine (*n* = 4, 11%), turkey (*n* = 6, 17%), chicken (*n* = 6, 17%), a chicken farm environment (*n* = 4, 11%), and human patients (*n* = 3, 9%). These isolates were collected from different locations in the United States during the period from 1999 to 2009. Isolates selected belong to Salmonella enterica serovars Heidelberg (*n* = 12, 34%), Typhimurium (*n* = 10, 29%), Newport (*n* = 6, 17%), Kentucky (*n* = 3, 9%), Anatum (*n* = 1, 3%), Dublin (*n* = 1, 3%), Cerro (*n* = 1, 3%), and Montevideo (*n* = 1, 3%).

Isolation approaches and phenotypic characterization of the isolates were described previously (5). We sequenced 35 Salmonella enterica isolates identified as carrying IncI1 plasmids using PCR-based plasmid replicon typing in our previous study (6). Each of the 35 isolates was stored at −80°C in brain heart infusion broth (Remel, Lenexa, KS) containing 20% glycerol, and prior to sequencing, isolates were subcultured on blood agar plates (tryptic soy agar with 5% sheep’s blood; Remel). All plates were incubated at 35°C for 24 hours. Overnight bacterial growth from individual isolates was scraped from the plate with a 1-μl inoculating loop and added to 180 μl animal tissue lysis (ATL) buffer (Qiagen, Valencia, CA, USA). Next, bacterial genomic DNA was extracted using a DNeasy blood and tissue kit (Qiagen). The quality and quantity of the DNA were examined using a NanoDrop instrument (Thermo Fisher Scientific, Grand Island, NY, USA) and a Qubit broad range (BR) assay kit (Thermo Fisher Scientific). DNA libraries were generated using 1 ng of DNA from each sample using the Nextera XT DNA library preparation kit (Illumina, San Diego, CA) and were multiplexed using combinations of two indexes of the Nextera XT index kit (Illumina). Isolates were sequenced in two batches with a maximum of 19 isolates per run. DNA sample libraries were diluted, denatured, and loaded onto the Illumina MiSeq instrument, and sequencing was performed using the v2 500-cycle kits. The two runs were monitored using a sequence analysis viewer with an emphasis on appropriate cluster densities of 1,193,000/mm^2^ with the final quality score (>Q30 score of 80.41) and 1,326,000/mm^2^ with the final quality score (>Q30 score of 80.66). FASTQ files were demultiplexed with MiSeq software, and the reads for each isolate were assembled using CLC Genomics Workbench ver. 9.0 (Qiagen, Redwood City, CA). Sequences were annotated initially using the Pathosystems Resource Integration Center (PATRIC) software version 3.5.36 (7). Subsequently, sequences were submitted to NCBI for final annotation through the Prokaryotic Genome Annotation Pipeline (PGAP) to annotate the draft genomes of these strains (8). The numbers of contigs, assembly sizes, coding sequences (CDS), and GC contents were annotated by PATRIC as shown in Table 1. The final annotations performed by the PGAP are available in NCBI under the accession numbers shown in Table 1.

**TABLE 1:** Whole-genome sequencing analyses of 35 Salmonella enterica isolates from food animal and clinical sources

Isolate	Serotype	Source	Yr of isolation	No. of sequence reads	*N*_50_ (bp)	No. of contigs	Genome length (bp)	No. of CDS	G+C content (%)	Accession no.
67	Newport	Cattle	2002	5,019,561	25,424	485	4,486,820	4,678	52.15	VCBN00000000
74	Newport	Cattle	2002	4,989,252	25,085	420	4,328,188	4,481	52.22	VCBO00000000
76	Newport	Chicken	2001	4,926,523	32,358	343	4,349,537	4,475	52.15	VCBP00000000
89	Newport	Swine	2001	4,977,554	26,443	407	4,620,558	4,755	52.18	VCIK00000000
93	Newport	Swine	2002	5,027,143	32,187	320	4,403,717	4,586	52.10	VCIL00000000
100	Newport	Turkey	2001	4,837,174	42,117	294	4,355,301	4,419	52.15	VCIM00000000
111	Heidelberg	Cattle	2001	5,182,383	29,076	363	4,895,560	5,076	51.95	VCIN00000000
114	Heidelberg	Cattle	2002	5,138,950	22,618	517	4,917,806	5,123	52.00	VCIO00000000
115	Heidelberg	Cattle	2002	5,191,943	45,514	263	4,657,342	4,803	51.96	VCIP00000000
116	Heidelberg	Cattle	2002	5,248,272	30,402	385	4,895,176	5,115	51.82	VCIQ00000000
159	Heidelberg	Turkey	2002	5,111,403	31,495	310	4,737,325	4,924	51.78	VCIR00000000
470	Typhimurium	Swine	1999	8,612,134	7,828	425	4,407,658	4,610	52.42	VCSK00000000
471	Typhimurium	Swine	1999	5,001,947	20,290	490	4,718,137	4,882	52.32	VCIT00000000
482	Typhimurium	Turkey	1999	4,960,480	24,216	417	4,670,043	4,763	52.22	VCIU00000000
695	Heidelberg	Turkey	2000	5,071,071	28,231	380	4,907,961	5,026	51.96	VCIV00000000
706	Heidelberg	Turkey	2000	4,897,846	25,101	405	4,522,077	4,649	52.13	VDBX00000000
715	Heidelberg	Turkey	2000	4,848,412	36,230	279	4,613,597	4,714	52.15	VCPT00000000
121	Heidelberg	Cattle	2002	4,846,037	36,611	379	4,843,682	5,006	52.23	VCSL00000000
N134	Typhimurium	Chicken farm	Unknown	5,086,478	92,540	130	4,508,660	4,683	52.00	VCQG00000000
N53	Typhimurium	Chicken	Unknown	5,049,701	19,622	577	4,912,152	5,091	52.23	VCQH00000000
849	Dublin	Cattle	2005	5,047,829	42,899	236	4,599,915	4,802	51.99	VCPU00000000
855	Typhimurium	Cattle	2006	5,014,625	49,906	226	4,499,875	4,632	52.05	VCPV00000000
856	Cerro	Cattle	2006	4,568,478	21,626	437	4,470,137	4,521	52.42	VCPW00000000
N865	Kentucky	Chicken	2008	4,879,802	23,260	503	4,523,952	4,615	52.08	VCQJ00000000
880	Montevideo	Cattle	2006	4,694,168	43,514	282	4,251,545	4,350	52.34	VCIS00000000
891	Anatum	Cattle	2006	4,741,580	28,637	300	4,601,255	4,657	52.18	VCPY00000000
990	Heidelberg	Human	2008	5,098,170	33,395	303	4,892,063	5,061	51.85	VCQK00000000
1000	Heidelberg	Human	2009	5,087,599	23,713	453	4,773,954	4,962	51.87	VCPZ00000000
1163	Heidelberg	Human	2007	4,950,681	49,656	344	4,678,168	4,854	52.01	VCQB00000000
N822	Kentucky	Chicken farm	2008	4,891,568	23,503	511	4,533,159	4,691	52.12	VCQI00000000
N860	Kentucky	Chicken	2008	4,880,247	27,698	417	4,486,160	4,589	52.02	VDBM00000000
N136	Typhimurium	Chicken farm	Unknown	5,082,919	41,441	275	4,700,886	4,820	52.02	VCQC00000000
N74	Typhimurium	Chicken	Unknown	5,099,545	35,108	326	4,587,257	4,780	51.88	VCQD00000000
N82	Typhimurium	Chicken farm	Unknown	5,056,644	22,986	497	4,740,914	4,928	52.12	VCQE00000000
N97	Typhimurium	Chicken	Unknown	5,054,428	36,102	279	4,729,717	4,859	52.04	VCQF00000000

Sequence data from each of the isolates were further analyzed using the PlasmidFinder (9) and ResFinder (10) tools to predict the presence of plasmids and antimicrobial resistance genes, respectively. PlasmidFinder analyses confirmed that all isolates contained IncI1 plasmids, many along with other plasmid replicon types, including IncA/C (*n* = 14, 40%), IncHI2 (*n* = 8, 23%), IncColpVC (*n* = 9, 26%), IncX1 (*n* = 7, 20%), and IncFIB (*n* = 6, 17%). ResFinder analyses were performed by selecting acquired antimicrobial resistance genes with default parameter settings. Multiple antimicrobial resistance genes, including *tet*(A) in 51% (*n* = 18) of the strains, followed by *bla*_CMY-2_ (*n* = 17, 49%), *aph(3ʺ)-Ib* (*n* = 16, 46%), *sul2* (*n* = 14, 40%), *fosA7* (*n* = 12, 34%), *aadA1* (*n* = 12, 34%), *floR* (*n* = 10, 29%), *sul1* (*n* = 9, 26%), *tet*(B) (*n* = 9, 26%), *bla*_TEM-1B_ (*n* = 8, 23%), *dfrA1* (*n* = 4, 11%), *bla*_TEM-1A_ (*n* = 1, 3%), *sul3* (*n* = 1, 3%), *dfrA2* (*n* = 1, 3%), *cml* (*n* = 1, 3%), and *cmlA1* (*n* = 1, 3%), were most commonly detected among the sequences. Default parameters were used for all bioinformatic software tools unless otherwise specified.

### Data availability.

This whole-genome shotgun project has been deposited at DDBJ/ENA/GenBank under the accession numbers listed in Table 1, and the SRA submission of the FASTQ files has been recorded under the accession number PRJNA543125.
